# Dr. Bevan Rake on Leprosy

**Published:** 1890-08-23

**Authors:** 


### MEDICINE.

### DR. BEAVEN RAKE ON LEPROSY.

The report for 1889 on the Trinidad Leper Asylum has recently reached this country. It is by Dr. Beaven Rake, the superintendent of the asylum. As Dr. Rake has long paid great attention to leprosy, and as he has the care in the asylum of some 170 lepers, his views may, at the present time, be read with interest. The burning question of the day, Dr. Rake states, is that of contagion. He cannot accept Father Damien's case, nor that of the inoculated convict Kearw, as absolutely proving contagion, for they both occurred in a country infested with leprosy. Living, as they were, in a colony of lepers, we cannot be sure whether they became infected by actual contact or from food, water, or some intermediary host. On this we may observe that several other cases have occurred, which, equally with the above, tend to prove the conveyance of leprosy by direct or indirect contact. Dr. Rake observes that most now believe in the leprosy bacillus, and the question is narrowed down to this: Can a healthy person more readily derive the leprosy bacillus from an infected human being, or from food, air, water, or some host which contains the bacillus or its spores? This question, however, cannot yet be answered, for we do not know the history of the bacillus outside the human body. It is quite possible that the bacillus may be conveyed both by direct communication and otherwise. Dr. Rake thinks that whether or not we accept Father Damien's and other cases as proving contagion, they become an argument for the segregation of lepers. We agree with Dr. Rake, so far as the segregation of lepers with open sores. But we cannot go further. We do not believe that leprosy is communicable until there are open sores. And we believe that a person with latent leprosy, or with leprosy very faintly developed, may yet have leper children. No system of segregation would prevent latent leprosy being hereditary. As showing the length of time leprosy may exist without becoming a severe disease, which would justify segregation, we refer to the case mentioned by Dr. Rake at page 9 of his report. A Hindu coolie had leprous spot on the arm for twenty-five years. With reference to the possibility of an intermediate stage of the leprosy bacillus, Dr. Rake examined the earth from the surfaces of eight graves in the cemetery at the leper asylum in which many bacilli were found. But similar bacilli were found in the earth over the body of a person who died of phthisis. In this connection it may be observed that Mr. Hutchinson has recently spoken of a bacillus common to both leprosy and tubercle, and Dr. Ransome has brought forward many items, showing a striking resemblance in other respects. We cannot, however, place much confidence in the discovery of bacilli in the earth, for Dr. Rake examined "earth from his garden a mile away from the asylum and found similar rods and staining there." With regard to food, Dr. Rake examined salt pork, salt fish, pigeon, peas, etc., but found no evidence of leprosy bacilli. Cultivation experiments also failed. With regard to the fish theory of leprosy, of which Mr. Hutchinson is the champion, the experience and experiments are decidedly adverse. And here we may mention the raised map of India by Mr. Hutchinson, displayed at the College of Surgeons, and professing to show by coloured lines, etc., the prevalence of leprosy in India. The coasts and lines of great rivers are dark, but there are other parts of India where there are no trees nor rivers, but where leprosy prevails to as great an extent in proportion to population. Yet these localities are left uncoloured, because, as we presume, there are no fish. Dr. Rake has inoculated cats, rabbits, pigs, guinea pigs, with negative results; but this does not prove anything, for such animals may not be susceptible of leprosy. Dr. Rake also gives a series of experiments on "protective and antagonistic inoculation." The material used was of different kinds, chiefly, however, tubercle, but the results cannot be regarded as satisfactory. Referring to treatment, the results from chaulmoogra oil are given. After months of use of this oil, Dr. Rake found increase of perspiration, decrease of tubercles, improved appetite, increase of sensation, lessening of pain. Chaulmoogra oil was first used for leprosy by the late Dr. Bhaw Dajee, of the Indian Medical Service, Bombay Presidency. It has been since extensively tried in India. We cannot, however, attribute improvement to the use of this oil alone. Even Bhaw Dajee said that it must be used many months. Now, the use of this oil for many months implies care of the leper in other respects. Being in a hospital he is under good hygienic and other conditions. Dr. Norman Chevers said that under good sanitation, good food, and the use of various oils, the anæmic leper became a robust leper, but remained a leper still. It must be remembered that in estimating the value of chaulmoogra oil or any other agent, that leprosy often improves spontaneously without any treatment, and still more frequently when generous diet is afforded. Dr. Rake also used creolin extensively. Creolin has been found to prevent the development of anthrax bacilli, and it was therefore considered it would have some deterring influence on leprosy bacilli. Dr. Rake says it reduces smell, promotes healthy granulations, and is about half the price of carbolic acid. Used internally, in the form of pills, no results were noted. Some remarks on the association of Addison's disease with leprosy follow, and a case of the kind is given. We cannot agree that the association is frequent, for the supra-renal capsules may be affected in leprosy as most other parts of the body may be. This does not, however, occur very often. Vandyke Carter states that all the supra-renal capsules examined by him in lepers were healthy. And it should not be forgotten that bronzing of the skin may occur without any affection of the supra-renal capsules. There is one passage in Dr. Rake's very interesting report which we do not understand. He observes, "Besides being a leper this patient was the subject of well-marked syphilis, as shown by the destruction of the nasal bones, the ulceration and loss of substance in the fauces and epiglottis, and the gumma in the liver."

On turning to a recent author on leprosy, we find it stated "Now the mucous membranes become affected. Hæmorrhages from the nose, mouth, or throat may occur, and there is much thickening deposit or ulceration in these structures, followed by destruction of bone, not unlike the ravages of tertiary syphilis. . . . Hoarseness and nasal voice are often symptoms of leprosy, and sometimes the nasal and palate bones are destroyed as in syphilis . . . neither would it appear that the tubercle of leprosy and the gumma of syphilis are very different in character, for they have a very close pathological resemblance." We, therefore, do not understand why Dr. Rake should rely on certain appearances as establishing the presence of one disease, when it is not to be denied that such appearances may be caused by another disease. Neither can it be ignored that some writers have regarded leprosy and syphilis as one and the same malady. For instance, Sir William Moore, in recent numbers of the *Provincial Medical Journal* and of the *Lancet*, forcibly advocates this view; relying both on intimate pathology and on the more superficial facts of resemblance of symptoms. Even those who do not adopt this extreme view have spoken of syphilitic leprosy, using such terms as leproid and syphiloid. The editor of the *Hospital Gazette* of June 4th last, in quoting Sir William Moore's article, observed, "In opposition to the views of most of our contemporaries, we have always maintained that leprosy is an exaggerated form of syphilis, and should be dealt with as such. The idea that it is caused by living too exclusively on fish diet or any other kind of food is all moonshine."

